# Information accumulation on the item versus source test of source monitoring: Insights from diffusion modeling

**DOI:** 10.3758/s13421-024-01636-2

**Published:** 2024-10-03

**Authors:** Hilal Tanyas, Julia V. Liss, Beatrice G. Kuhlmann

**Affiliations:** Department of Psychology, School of Social Sciences, University of Mannheim, Mannheim, Germany

**Keywords:** Source monitoring, Source memory, Item memory, Diffusion model, Temporal sequence

## Abstract

Source monitoring involves attributing previous experiences (e.g., studied words as items) to their origins (e.g., screen positions as sources). The present study aimed toward a better understanding of temporal aspects of item and source processing. Participants made source decisions for recognized items either in succession (i.e., the standard format) or in separate test blocks providing independent measures of item and source decision speed. Comparable speeds of item and source decision across the test formats would suggest a full separation between item and source processing, whereas different speeds would imply their (partial) temporal overlap. To test these alternatives, we used the drift rate parameter of the diffusion model (Ratcliff, *Psychological Review*, *85*, 59–108, 1978). We examined whether the drift rates, together with the other parameters, assessed separately for the item and source decision varied as a function of the test format. Threshold separation and nondecision time differed between the test formats, but item and source decision speeds represented by drift rates did not change significantly. Thus, despite facilitation on the source decision when the item decision was immediately followed by a test for source memory than when item and source were tested in separate blocks, findings did not suggest that source information already begins accumulating in the item test in the standard format. We discuss the temporal sequence of item and source processing in light of different assumptions about the contribution of familiarity and recollection.

**Supplementary Information:**

The online version contains supplementary material available at 10.3758/s13421-024-01636-2.

## Introduction

Do we first remember a piece of information itself (i.e., item) and then its context features (i.e., source) – for example, where, when, or how we learned it – or can the retrieval of both overlap to some extent? Three decades ago, Johnson et al. (1993) introduced the source-monitoring framework and outlined the set of memory and judgment processes involved in attributions of mental experiences to their sources. Accordingly, both recognizing previous experiences (item memory, i.e., old or new?) and identifying their contextual details (source memory, i.e., source A or source B?) can be described within the source-monitoring framework with varying levels of differentiation. While item recognition succeeds even at lower differentiation levels, source attribution relies on more complete information. The objective of the current study was to test the temporal sequence of item and source processing more closely. Specifically, we were interested in whether source processing already starts in parallel to item processing or only starts sequentially to the successful item retrieval.

To illustrate that less differentiated information becomes available at earlier stages of processing, Johnson et al. (1994) investigated the time-course functions for item and source memory in an internal-external source-monitoring paradigm, also referred to as *reality monitoring* (Johnson & Raye, 1981), assessing memory for imagined versus perceived items. They employed the response-signal technique (Reed, 1973, 1976) and manipulated the amount of time allowed for retrieval systematically across varied response lags in a test where item and source judgments were collected simultaneously (response options: “imagined,” “perceived,” or “new”). This is an established experimental method that was also applied in earlier time-course studies investigating the temporal availability of context information (e.g., Dosher, 1984; Dosher & Rosedale, 1991; Hancock, 2002). Concerning its relevance to the focus of interest, the response-signal technique was employed to compare the time course of item recognition with that of modality recognition (a source-memory task, see Hintzman & Caulton, 1997) or associative recognition (Gronlund & Ratcliff, 1989), differing from source memory with nuances in which each item is paired with another item (not a source) at study. Both studies revealed earlier availability of item information. Crucially, Johnson et al. (1994) further benefited from model-based approaches considering the multitude of processes involved in source-monitoring decisions. They assessed item and source memory separately and corrected for guessing based on multinomial-model parameters (Batchelder & Riefer, 1990). Consistent with the source-monitoring framework, the results suggested earlier accessibility of item information than source information. In subsequent years, more rigorous tests on Johnson et al.’s (1994) data (Kinjo, 1998; McElree et al., 1999) raised concerns regarding the conclusiveness of the original findings. Following that, Kinjo (1998, Experiment 1) conducted a stronger test in a modified procedure with more response-lag conditions and still observed that item memory was accessed before source memory. Later on, Spaniol and Bayen (2002) also used the combination of multinomial modeling (Bayen et al., 1996) and the response-signal technique. However, the authors compared the time-course functions of item memory and source *guessing bias* (but not source memory). They observed that item memory became available before source guessing.

Measurement models characterizing the underlying processes of item and source judgments with different assumptions (e.g., threshold models vs. signal-detection models; see Bayen et al., 1996, and DeCarlo, 2003, respectively) also fostered the time-course research indirectly by probing discussion on whether there is source memory for unrecognized items (e.g., Malejka & Bröder, 2016; Starns et al., 2008; see also Fox & Osth, 2022, for an overview). However, these models do not posit a temporal ordering between the memorial information. Specifically, even though the two-high threshold multinomial model of source monitoring (Bayen et al., 1996) postulates source discrimination as contingent on successful item recognition, the order of the item and source memory parameter in the multinomial model branches does not postulate a serial ordering. Rather, both could occur simultaneously in these branches (cf. Batchelder & Riefer, 1999). Indeed, Johnson et al. (1994) underlined this possibility of the parallel retrieval of item and source information under the serially represented structure of the multinomial model of source monitoring. Further, the source-monitoring framework predicts that differentiation of different memory characteristics can occur at different rates. However, it does not claim a full separation such that the completion of one processing is necessary for the onset of another processing. Instead, their time course can closely intertwine (see Fig. 1B in Johnson et al., 1993).

Interestingly, this possible alternative of parallel processing of item and source information is completely unaddressed by the published literature. Even though it can be concluded from direct investigations of response time lags that source retrieval completes later than item retrieval (cf. Johnson et al., 1994), it is not possible to ascertain from combining the response-signal technique and multinomial modeling whether source information is retrieved serial to completed item processing or already started in parallel to item processing (i.e., item processing starts before source processing, but their retrieval courses overlap to some extent). More broadly speaking, time-course curves of the response-signal technique display the change of accuracy as a function of response time characterized by separate parameters, thus allowing us to measure whether the onset of one processing occurs before the onset of another processing. Yet, under this particular circumstance, these time-course curves are described for parameter estimates of memory processes, and memory accuracy is measured with multinomial threshold models. Crucially, these specific analyses only tap into whether source processing accumulated sufficiently to cross the source-discrimination threshold but cannot indicate whether it was already started during earlier stages. Further, restricting the time available for responding may increase the risk of altering the cognitive processes and, in particular, their mental organizations. Although the response-lag technique is an insightful temporal study design, we still deem it important to further pursue this line of research with different methods. In contrast to the extensive investigation of item and source accuracy performance, for example with experimental dissociations (e.g., Lindsay & Johnson, 1991), research on the *temporal* aspects of item and source processing is relatively scarce, and to our knowledge, has only been conducted directly with the response-signal technique thus far. Therefore, to fully explore the breadth of the time-course question, we should expand our analysis to include spontaneous (i.e., not temporally restricted) source retrieval and, thereby, consider promising alternative methodologies.

Recently, Tanyas and Kuhlmann (2023) tried to address the question of the seriality versus partial overlap of item and source memory with the mouse-tracking method (cf. Kieslich et al., 2019), which allows us to measure these retrieval processes dynamically as well as to outline their temporal development. The item (old or new?) and source tests (source A or source B?) were presented either consecutively for each recognized item (i.e., directly following each “old” response) as in the standard research of source monitoring, or the source test of the recognized items was presented as a separate block after the full completion of the item test (for a similar blocked test procedure in source monitoring, see Osth, Fox, et al., 2018a). Contrary to the blocked format which provides relatively more independent measures of item and source (serving as the baseline), participants made their source decisions more straightforwardly than their item decisions in the standard format as evidenced by the smoother (less curved) source trajectories than the item trajectories. There are two alternative interpretations of this pattern. First, during the item test of the standard format, source information might have been retrieved parallel to item information as preparation for the ensuing source test. Second, rather than parallel processing, being already in the item recognition state might have rendered source retrieval more accessible. Carefully note that in the second scenario, source retrieval can be assumed to have operated in sequence, rather than in parallel, to item retrieval and still can explain the observed difference in the source trajectory pattern. Thus, the observed movement trajectories during the source tests are not conclusively indicative of serial or parallel item versus source processing. It may seem that the temporal sequence of item and source processing is far from being resolved, but these results clearly underline the close links of item and source retrieval courses. Next steps will be to consider different techniques that are better suited to a closer look at this fine-tuning association.

To conclude, a more thorough examination is needed to capture important nuances which might underly response times (RTs) and mouse trajectories in source-monitoring processes. Notably, responses from such a higher-order cognitive task as source monitoring may reflect different processes of which only some are relevant to the item versus source attribution specifically. To disentangle these latent processes, the diffusion model is a promising candidate and may open up new avenues to the time-course question in source monitoring.

### Diffusion modeling in episodic memory research

**Fig. 1:**
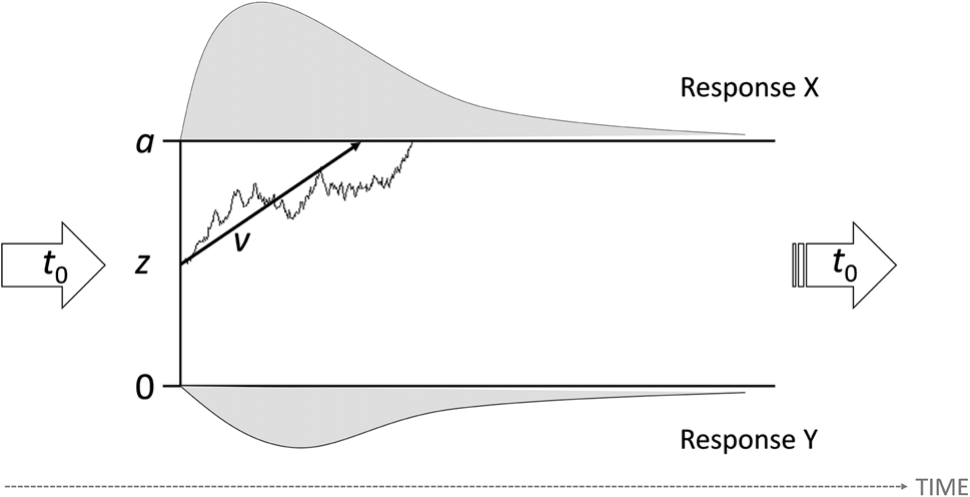
Illustration of the decision process as proposed by the diffusion model (Ratcliff, 1978). Here, the upper and lower thresholds correspond to decisional outcomes (alternatively, correct and incorrect responses). The distance between the thresholds is represented by *a*. Information accumulation starts at *z* (here centered between the two thresholds) and continues over time with speed |*v|* (denoted by the upward pointing arrow) until it reaches either of the two response alternatives. Random influences lead to unsteady fluctuations in the sample path. The duration of processes outside the decision process (e.g., encoding or response execution) are accounted for *t*_0_. The response time distribution for choosing response X (response Y) is shown above (below) the respective threshold

When considered from the traditional viewpoint of cognitive psychology, mean RT performance is considered the index of mental chronometry (cf. Balota & Yap, 2011). As a consequence of this approach, information from a number of experimental trials is condensed into a single mean, resulting in loss of information and a missing common metric that also accounts for accuracy. The diffusion model (Ratcliff, 1978) is highly recommended to overcome these problems because it includes full distributions of RTs of correct and incorrect responses (e.g., Vandekerckhove & Tuerlinckx, 2007; Voss et al., 2013; Wagenmakers, 2009). It assumes that during a binary choice task, information accumulates continuously until one of two thresholds (i.e., alternative decisional outcomes) is reached (see Fig. 1). This decision process is driven by systematic and random influences. Based on the RT and accuracy data from all test items, the model provides separate parameters for the speed of information accumulation (i.e., the drift rate, parameter *v*), the amount of information considered in decision making (i.e., threshold separation, parameter *a*), possible a priori decision biases (i.e., starting point, parameter* z*), and the duration of nondecisional processes (e.g., encoding and response execution, parameter *t*_0_). In addition to these four key parameters, other parameters have also been added to the model over time such as to account for intertrial variability (i.e., parameters *s*_*v*_, *s*_*z*_, *s*_*t*0_; see Ratcliff & Rouder, 1998; Ratcliff & Tuerlinckx, 2002) and differences in speed of response execution (parameter *d*; see Voss et al., 2010). Overall, the diffusion model allows researchers to understand whether – and especially in what ways – task performance can be explained by psychologically meaningful processes (Voss et al., 2013).

One important domain for the diffusion model is episodic memory tasks on which item recognition is assessed in binary response options. In his seminal study, Ratcliff (1978) introduced the diffusion model on the recognition memory paradigm. It then became a useful tool in recognition studies for several reasons such as to decompose age-related changes (e.g., McKoon & Ratcliff, 2012; Ratcliff et al., 2004, 2011, Ratcliff & McKoon, 2015; Spaniol et al., 2008), to investigate emotion-modulated memory (Bowen et al., 2016) and clinical disorders (White et al., 2010), and to enhance understanding of the strength-based mirror effect (Starns et al., 2012). The theoretical assumptions of the diffusion model are thus well met in recognition tests (Voss et al., 2013), and Arnold et al. (2015) further showed empirical validity of the diffusion model parameters for recognition memory. Most relevant to our research goal, Spaniol et al. (2006, Experiment 2) extended the use of the diffusion model to a two-choice source-monitoring task (also see Starns, 2014, for another application of the diffusion model to a source-memory task) to separately estimate the contributions of different processes to episodic source retrieval in younger and older adults. They interpreted the drift rate in the source task as “the quality of the contextual information driving the decision process during retrieval” (p. 116). Importantly, the drift rate *expectedly* showed age differences in episodic but not semantic memory tasks, meaning that it was sensitive to the specific memory processing of interest. Inspired by this extension, here we employed the diffusion model to separate cognitive processes underlying both item and source decisions on the parameter level and, in particular, to better capture item versus source processing speed with the drift rate estimates.

### The current experiment

In the standard sequential test of source monitoring (cf. Lindsay et al., 1991), item recognition for each trial is immediately followed by a source test. We aimed herein to investigate whether source decisions are reached after item decisions, compatible with this order of testing, or whether there can be some temporal overlap in item and source processing such that the latter is already started during the first item test step. Similar to the rationale of Tanyas and Kuhlmann (2023), we manipulated different test formats of source monitoring so that item and source information were either tested in immediate succession (i.e., the standard format) or temporally separated (blocked) for the recognized items (i.e., hits and false alarms). Thus, both test formats include an item test for all stimuli and a source test for items only judged as old. Critically, in the standard format, participants were informed in advance that they would be tested for the source immediately following each item recognition. In the blocked format, however, participants did not have prior knowledge about the upcoming source test block, and they were instructed to focus only on their item decisions during the item test block. Consequently, the blocked test format would provide a relatively more independent measure of item versus source decision speeds because the item and source tests are separated in time; thereby, they are more informative specifically about the duration of item versus source processing. Differences in decision speeds in the standard format with reference to the blocked format would then be informative regarding whether participants retrieved item and source in a sequenced or in a (partially) parallel way.

Of interest were RT data in relation to the accuracy of item and source test responses and parameter estimates for the diffusion model derived therefrom. We used the absolute values of parameter *v* as a measure of decision speed (of item vs. source processing, respectively) in each test. The faster the information accumulation, the higher the absolute drift rate. Carefully note that in comparisons across task conditions the drift rate maps onto task difficulty, such that easier tasks are associated with higher absolute drift rates (Lerche & Voss, 2019; Ratcliff & McKoon, 2008; Voss et al., 2004). Based on higher differentiation and greater recollection demands in source memory (Johnson et al., 1993; Yonelinas, 1999), slower speed of information accumulation in the source test (parameter *v*_source_) compared to the item test (parameter *v*_item_) could be expected. Most importantly, however, we planned to compare the speed of one type of processing (i.e., item or source) with its pendant between the test formats. Our following hypotheses explain how these test format comparisons can inform us about the seriality or parallelism of item versus source processing:

#### H1

If we observe statistically comparable item and source decision speeds across the standard and the blocked test formats (i.e., no interaction of test format and memory type), this suggests a full separation (or temporal sequence) between item and source processing. Notably, in the blocked test format, while participants are responding old/new, they do not know yet whether (and when) there will be a test for source at all. Put differently, we do not give participants the chance to benefit from parallel retrieval of item and source in the item test block. Therefore, if item and source processing are sequential in the standard format, the speeds (i.e., of item and source) should always be the same as in the blocked format because this would always mean that the item is processed with its speed and subsequently the source is processed with its speed.

#### H2

By contrast, if item and source decision speeds differ by test format, our inference about temporal overlap would be based on the specific direction of condition differences. We would most plausibly expect the transfer of part (or all) of information accumulation from the source test of the standard format to its item test, which should be represented by slower item drift rates in the standard format compared to the blocked format. This would then suggest that source processing already started during the item test of the standard format. Consequently, we would also expect faster source processing in the standard format than the blocked format, indicating that part of the information accumulation in the source test must have been outsourced to the item test of the standard format.

As preregistered, we additionally explored whether the other parameters of the diffusion model also differ across the test formats in order to gain a better understanding of what composes the full RT distributions of item and source decisions in the standard sequential source-monitoring test.

## Method

All materials and data together with our preregistration protocol are available online on the Open Science Framework. The preregistration protocol is available at https://osf.io/j9zwr/registrations. The experiment script and the results (including the supplementary analyses) are openly available from https://osf.io/j9zwr/.

### Participants

A power analysis for an *F* test conducted with the G*Power-3 software (Faul et al., 2007) indicated that a sample size of 30 per test format condition (*N* = 60) would provide a power of .80 to detect a medium-sized (*f* = .25) interaction between memory type and test format (α = .05, correlation among repeated measures = .10; see also Tanyas & Kuhlmann, 2023, for a similar logic). As explained in our hypotheses, the detection or rejection of this interaction is most relevant to deciding on the seriality versus parallelism of item and source processing.

Inclusion criteria to participate in the study were native fluency in English (learned before the age of 6 years); age (18–30 years); normal or corrected-to-normal vision; no diagnosis of mild cognitive impairment; no mental illness daily impact; no head injury that caused a knock-out for a period of time; no severe respiratory diseases (i.e., pneumonia or chronic obstructive pulmonary disease (COPD)); no medically diagnosed coronary artery or heart issues; no use of medication affecting cognition. We used Prolific’s prescreening filters and additionally checked for these criteria before allowing participants to complete the full study. For the completed datasets, we also preregistered performance-based exclusion criteria that all participants should perform above-chance item memory (i.e., Hit rates > False alarm rates) and above-chance source memory (i.e., ACSIM score (average of the single-source conditional source identification measures, CSIM; cf. Murnane & Bayen, 1996) above .50). The reasoning behind that was that memory should drive most of the responses in the tests such that the drift rates tap into the speed of item versus source *memory* specifically. Thus, we recruited a total of 80 participants from the online recruitment platform Prolific (https://www.prolific.com/; also see Palan & Schitter, 2018) to meet the goal of analyzing data from a total of 60 participants. Nineteen participants were excluded from the data because they did meet the performance-based exclusion criteria. One participant took the study twice because of technical/internet problems and thus was also excluded. As reported later, we removed data from three participants based on our diffusion model analyses (see section Parameter estimation and model fit). Thus, the results reported are based on 57 participants (33 female, 23 male, one preferred not to indicate sex; *M*_age_= 25.47 years, age range = 19–30 years). The experiment lasted approximately 30 min. Participants received payment according to the Prolific-set rate of £6/h.

### Design

The design was a 2 (test format: the standard format vs. the blocked format) × 2 (memory type: item memory vs. source memory) mixed factorial design with memory type as a within-subjects and test format as a between-subjects factor. We also manipulated spatial position of study words (top vs. bottom of the screen) serving as the source manipulation as a within-subjects factor. However, we expected comparable item and source memory across sources and aggregated (as already planned in our preregistration) across spatial position for analyses (see Online Supplementary Material (OSM)).

### Materials

We randomly selected 108 English nouns from the Toronto Word Pool (Friendly et al., 1982) after controlling for certain characteristics with the goal of selecting memorable items (imagery: ≥ 1.5 on a 7-point scale, concreteness: ≥ 2 on a 7-point scale, and Kucera-Francis frequency: ≥ 20). From this set, assignment of the words as study items (72 words) and distractors (36 words) as well as assignment of study items to the sources (50% on the top vs. the bottom of screen) were randomized anew across participants.

### Procedure

We recruited participants on the platform Prolific, pre-filtering in accordance with our inclusion criteria. After seeing a detailed description and requirements of our study on Prolific, participants were redirected to OpenLab (https://open-lab.online/; Shevchenko, 2022) for the experimental task, which was programmed in an online study builder lab.js (based on HTML and JavaScript; see Henninger et al., 2022). The assignment to the test format conditions (the standard format vs. the blocked format) was randomized by OpenLab’s urn function. After consenting, participants completed a demographic and health questionnaire, and we made sure that their responses were matched with Prolific’s prescreening filters and thus checked our inclusion criteria again. If participants were deemed not eligible to participate, the session was terminated, and they received partial payment.

Eligible participants continued with the source-monitoring task. To increase memory-based responses in the later test, instructions emphasized before study that participants should learn both words (items) and their screen positions (sources) and that they would be informed later which exactly they will be tested on (cf. Tanyas & Kuhlmann, 2023). Before studying words, participants saw two fixed primacy buffer items (one on the top and one on the bottom with a randomized order for each participant) but not as part of the words used in the source-monitoring test, and later they were presented in the practice test again along with two more distractor words. During study, 72 words were presented either on the top or on the bottom of the screen (random assignment of half of the items to each position) in a pseudorandom order with the restriction that there were no more than three consecutive repetitions in the same screen position. Each study item was shown in the respective position for 4 s, separated by a 500-ms inter-stimulus interval (a centered fixation cross and a blank screen, each lasting for 250 ms). Next, as a filler activity, participants verified simple math equations for 3 min. Finally, participants completed a self-paced source-monitoring test, designed according to their assigned test format condition. All stimuli were printed with 36-pt (corresponding to 48 px) Arial font in black against a white background throughout the experiment.

**Fig. 2:**
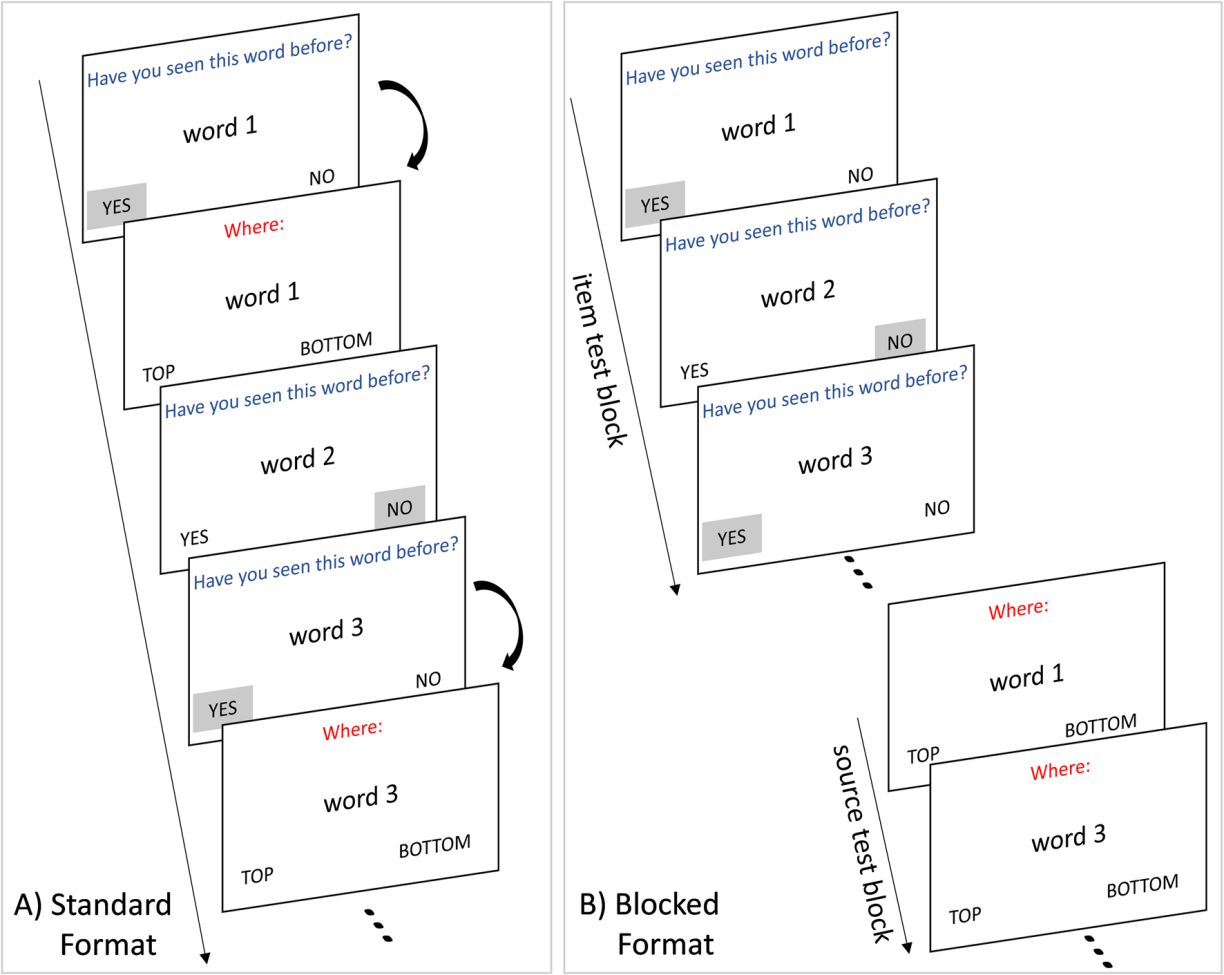
Example visualizations of the test formats. (**A**) In the standard format, source decisions for each recognized test trial were collected in immediate succession to item decisions. (**B**) In the blocked format, source decisions for all recognized test trials were collected as a separate test block after the completion of the item tests

Participants in the standard format were informed that during their item decisions, if they indicated that a test trial was shown in the study phase before, their source memory for that trial would be tested immediately after (see Fig. 2A). In the blocked format, however, before the test session, participants were (truthfully) informed that only the words (not positions) matter for the responses here. We did this to maintain item test validity, as reasoned in the Introduction section. Thus, in this condition, participants were first questioned about whether the test trials were shown in the study phase or not, without being provided any information about the upcoming source test block yet. After the completion of the item test for all test trials, participants were then retested on the words they had judged to be “old” in the same order as on the item test and asked to indicate their studied positions (see Fig. 2B).

Participants in all conditions were instructed to respond as accurately and as fast as possible. At test, they were presented with a list that consisted of the items from both sources and new items (i.e., distractors), but this time all appeared centered one at a time. During the item test, the question “Have you seen this word before?” appeared in blue on the upper portion of the computer screen above the test trials with the two response options. On the keyboard, “left arrow key” and “right arrow key” were assigned as “YES” and “NO,” respectively. During the source test, however, the previous question was replaced by the word “Where:” and appeared in red, and both source options appeared side by side on the screen. In order to indicate source decisions, participants were required to press “up arrow key” or “down arrow key,” representing “TOP” or “BOTTOM”, respectively. These answer choices and key assignments were shown again on the test screen. We told them to simply guess if they could not remember whether and/or where the word was presented. Note that we needed targets to be able to assess source attributions, and more distractors are not particularly informative for our research question as for them a source cannot be retrieved. Therefore, we kept the same number of words for the categories “top,” “bottom,” and “new” (i.e., 36 for each). The presentation order of the test trials was also randomized by participants. Our lab.js scripts recorded response accuracy and RTs automatically.

## Results

### Parameter estimation and model fit

For the item test, we included both targets and lures in the analyses, and the thresholds of the diffusion model were linked to actual responses. Therefore, the upper and lower thresholds stand for “old” and “new” responses, respectively. However, for the source test, we restricted our analyses to only the items correctly identified as “old” because there cannot be source memory for lures as they were never presented with a source. The thresholds of the diffusion model were mapped to response accuracy. Thus, in the source test, the thresholds correspond to correct and incorrect source attributions given upon correct target detections. Considering the small trial number in our data, we used the maximum likelihood optimization criterion but with a strict outlier elimination procedure (following Lerche et al., 2017; Voss et al., 2013). Responses faster than 100 ms or slower than 4,000 ms were excluded from analyses (cf. Spaniol et al., 2006; Whelan, 2008). As an individualized elimination method, we additionally applied Tukey’s outlier criterion (Tukey, 1977) separately for the item (considering the RT distribution based on both targets and lures) and source RTs to discard further possible contaminants. We removed trials that were more than three interquartile ranges below or more than three interquartile ranges above the third quartile of a participant’s log-transformed RT distribution (e.g., Lerche, Neubauer, et al., 2018b). Prior to all analyses, we thus excluded a total of 2.21% of trials across participants.

Using the software fast-dm (Version 30.2; Voss & Voss, 2007; Voss et al., 2015), we fitted the diffusion model separately to each participant’s data from the item test and the source test using the maximum likelihood optimization criterion. More specifically, for the item test, parameters comprised drift rates (*v*) for targets and lures, threshold separation (*a*), nondecision time (*t*_0_), and relative starting point (*z*_*r*_ = *z*/*a*; *z*_*r*_ > 0.5 represents a bias towards the “old” response, whereas there is a bias towards the “new” response if *z*_*r*_ < 0.5). Given the sensitivity of the maximum likelihood optimization criterion towards fast outliers, we additionally estimated the intertrial variability of nondecision time (*s*_*t*0_) to possibly avoid the negative effects of fast contaminants and to improve the estimation of the main parameters (Lerche & Voss, 2016). The intertrial variabilities of drift rate and starting point (*s*_*v*_ and *s*_*zr*_), however, were fixed at zero for the sake of model parsimony and due to challenges associated with their estimation (Boehm et al., 2018; also see van Ravenzwaaij et al., 2017).

Note that the source test was conditional on the item test (i.e., an “old” response), indicating that the source trials entering the analyses were fewer than the item trials. We thus kept the model as simple as possible for the source test due to the restricted trial numbers (e.g., Lerche, Neubauer et al., 2018b; also see Lerche et al., 2017). More specifically, the upper and lower thresholds corresponded to correct and incorrect responses, respectively, and we assumed the relative starting point to be unbiased, fixing it at .5 (cf. Voss et al., 2013). Consistent with the item test, for the source test, we estimated *s*_*t*0_ once per participant and set *s*_*v*_ and *s*_*zr*_ to zero.

In total, we estimated ten parameters per participant (i.e., *v*_itemtargets_, *v*_itemlures_, *v*_source_, *a*_item_, *a*_source_, *t*_0item_, *t*_0source_, *z*_*r*item_, *s*_*t*0item_, and *s*_*t*0source_).^1^ Two participants had to be excluded because parameter estimates could not be obtained due to very few source trials. One participant had a high proportion of too slow item responses rendering the parameter estimation difficult and was also removed from the results. We report the following analyses based on the remaining 57 participants.

An acceptable model fit is a prerequisite for analyzing and interpreting the diffusion model parameters. Figure 5 in the Appendix shows a graphical evaluation of model fit separately for the test formats by means of scatter plots. These scatter plots compare the accuracy rate (i.e., proportion of correct responses out of the total number of responses) and several RT quantiles of the behavioral data against the corresponding statistics predicted by the diffusion model based on the parameter estimates. The empirical and predicted values are described along the *x*- and *y*-axes, respectively. Each data point thus reflects one participant, and the discrete symbols accompanied by different colors refer to the item (along with item type) and source tests. Overall, data points are positioned tightly on or near the plots’ main diagonal, indicating that the diffusion model provided a good account of the data of both groups.

### Analyses of behavioral variables

**Fig. 3:**
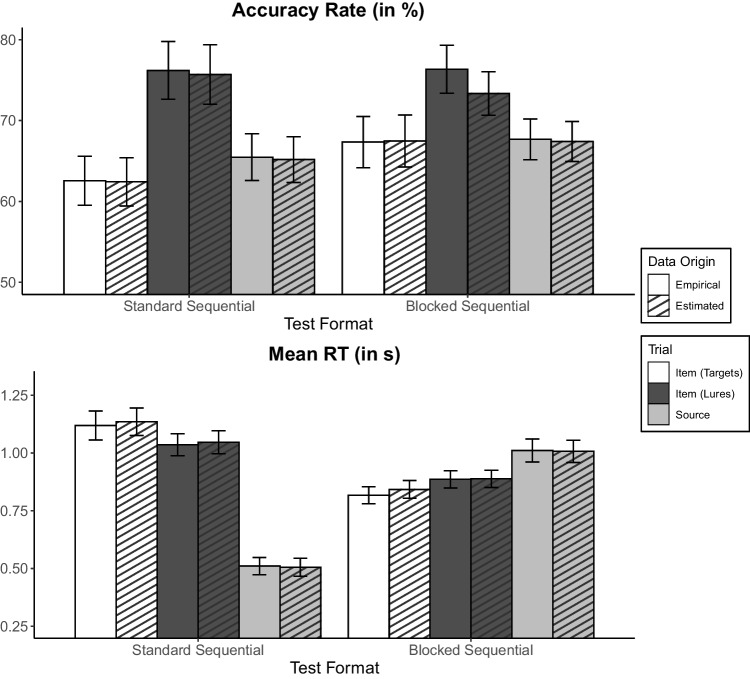
Empirically observed mean accuracy rate and correct response times (RTs) across conditions with their estimated counterparts. Error bars represent standard errors. Mean RT = mean response time of correct responses

Before further examining the diffusion model’s parameter estimates, we first report empirical statistics for the behavioral variables’ accuracy rate and mean RTs for the 57 participants included in the analyses. Mean accuracy rate and correct RTs are given in Fig. 3. Restricting to targets only, we performed separate 2 × 2 mixed ANOVAs using accuracy rate and mean RTs for correct responses with the within-subjects factor memory type and the between-subjects factor test format. The alpha level was set at .05, and we report partial eta squared (${\upeta }_{\text{p}}^{2}$) as the measure of effect size.

For accuracy rate, neither the main effects of test format nor memory type nor their interaction were significant (all *F*s < 1). Because accurate test responses may stem from memory or guessing, we also applied the multinomial processing tree (MPT) model of source monitoring (Bayen et al., 1996) to the present data as a more comprehensive analysis of the processes involved. Interested readers can find these more fine-grained accuracy analyses in the OSM.

In the analysis of correct mean RTs, the main effect of memory type, *F*(1, 55) = 37.22, *p* < .001, ${\upeta }_{\text{p}}^{2} =$.40, and the test format × memory type interaction, *F*(1, 55) = 138.48, *p* < .001, ${\upeta }_{\text{p}}^{2}=.72$, were significant, but not the main effect of test format, *F*(1, 55) = 2.94, *p* = .092, ${\upeta }_{\text{p}}^{2} =$.05. Relevant to our interest, the simple main effect analyses following up on the significant interaction revealed that correct mean RTs in the item test of the standard format were slower than in the item test of the blocked format, *F*(1, 55) = 18.13, *p* < .001, ${\upeta }_{\text{p}}^{2} =$.25. In contrast, correct mean RTs in the source test of the standard format were faster than in the source test of the blocked format, *F*(1, 55) = 62.70, *p* < .001, ${\upeta }_{\text{p}}^{2} =$.53.

Next, we describe our subsequent statistical analyses on individual estimates of the diffusion model parameters. Thereby, we can test whether the observed effects on item and source RTs reflect changes in the actual processing of item and source decision and/or in nondecisional aspects of the test responses (e.g., the motoric response).

### Analyses of model parameters

**Fig. 4:**
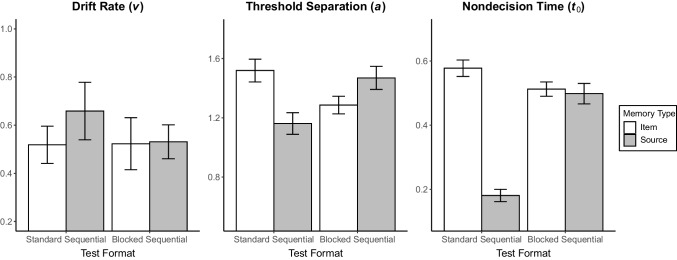
Bar plots of the diffusion model parameters drift rate, threshold separation, and nondecision time. Error bars represent standard errors. For drift rates, absolute values are shown

**Fig. 5:**
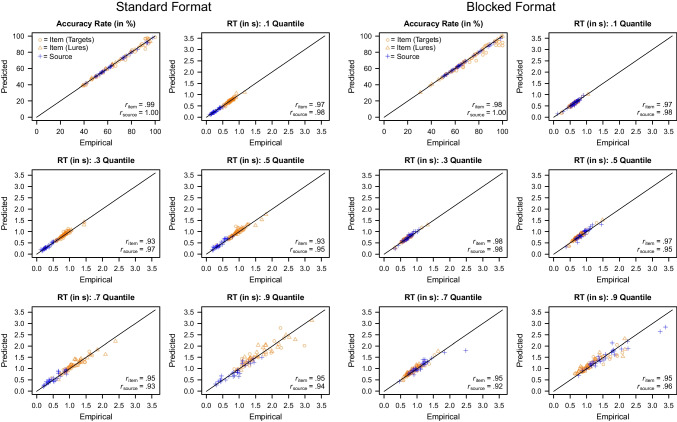
Graphical displays of model fit. Concordance of the empirical and predicted statistics for the accuracy of responses and the .1, .3, .5, .7, and .9 quantile of correct response time distributions for each person in each condition*.* RT = response time; *r*_item_ = correlation between empirical and predicted statistics for item test; *r*_source_ = correlation between empirical and predicted statistics for source test

Means of the estimates of the diffusion model parameters for conditions are presented in Table 1. Here, we examined three main diffusion model parameters (i.e., *v*, *a*, *t*_0_) as the dependent variables and report inferential statistics on their estimated values. We conducted separate mixed ANOVAs using the individual parameter estimates of participants with the within-subjects factor memory type and the between-subjects factor test format. Bar plots for all three examined parameters are given in Fig. 4.

**Table 1:** Group mean estimates of the diffusion model parameters and the *t* tests for the comparison of test format

	Test format		*t* test		
Parameter	Standard format (*n* = 27)	Blocked format (*n* = 30)	*t*(55)	*p*	Cohen’s* d*
*v*_itemtargets_^a^	0.52 (0.08)	0.52 (0.11)	0.03	.975	0.01
*v*_itemlures_^a^	0.94 (0.15)	1.27 (0.12)	1.73	.089	0.46
*v*_source_^a^	0.66 (0.12)	0.53 (0.07)	−0.94	.349	−0.25
*a*_item_	1.52 (0.08)	1.29 (0.06)	−2.42	.019	−0.64
*a*_source_	1.16 (0.07)	1.47 (0.08)	2.87	.006	0.76
*t*_0item_	0.58 (0.03)	0.51 (0.02)	−1.92	.060	−0.51
*t*_0source_	0.18 (0.02)	0.50 (0.03)	8.36	< .001	2.22
*z*_*r*item_	0.48 (0.02)	0.58 (0.02)	3.67	< .001	0.97
*s*_*t*0item_	0.22 (0.03)	0.14 (0.03)	−1.98	.052	−0.53
*s*_*t*0source_	0.07 (0.02)	0.16 (0.03)	2.24	.029	0.59

*Note.* Standard errors are presented in parentheses

^a^Prior to analyses, we calculated the absolute values of drift rate estimates to allow for the comparison of drift rates in terms of absolute size

Drift rate (parameter *v*) indicates the direction and speed of information accumulation across all trials. Its sign is positive if the diffusion process reaches the upper threshold in the majority of trials and negative otherwise. Absolute drift rates, however, capture the speed of information accumulation independent of its correctness, with higher values representing faster accumulation (cf. Lerche & Voss, 2016). Our main interest was to understand whether participants already made part (or all) of the information accumulation for the source decision during the item test of the standard format (regardless of whether their decision processes mostly reached the correct or incorrect threshold).^2^ We thus submitted the absolute values of drift rates for the item (again, only targets) and source test to the 2 × 2 ANOVA. Neither the main effects of test format, *F* < 1, nor memory type, *F*(1, 55) = 1.18, *p* = .282, ${\upeta }_{\text{p}}^{2} =$.02, were significant. Importantly, test format did not interact with memory type, *F* < 1. This pattern is in line with our preregistered H1 supporting temporal sequence of item and source processing in the standard format. Item and source decision speeds did not differ by test format, such that the speed of information accumulation was statistically comparable when tested in succession or a blocked manner. Thus, there was no convincing evidence for a transfer of source processing to the item test in the standard format. In the following, we report additional exploratory analyses on the other diffusion model parameters.

Threshold separation (parameter *a*) is informative for how much information is required to decide on a response. For threshold separation, there was no significant main effect of test format, *F* < 1. However, the main effect of memory type, *F*(1, 55) = 5.39, *p* = .024, ${\upeta }_{\text{p}}^{2} =$.09, and the test format × memory type interaction were significant, *F*(1, 55) = 51.32, *p* < .001, ${\upeta }_{\text{p}}^{2} =$.48. Follow-up simple main effects analyses revealed that a larger amount of information was needed for an item response in the standard format compared to the blocked format, *F*(1, 55) = 5.88, *p* = .019, ${\upeta }_{\text{p}}^{2} =$.10. Notably, the respective difference was reversed in the source test such that less information was required in the standard format than in the blocked format, *F*(1, 55) = 8.21, *p* = .006, ${\upeta }_{\text{p}}^{2} =$.13. As threshold separation is part of the item and source decision making of interest to us, this pattern supports facilitation on the source decision by prior item retrieval (not significant for accumulation as captured by drift rates but significant for threshold).

Nondecision time (parameter *t*_0_) estimates the remaining time outside the diffusion process such as encoding stimulus and execution of the motor response. It is thus not informative about item and source processing per se but about differences in these additional demands between the two test formats. For nondecision time, the main effects of test format, *F*(1, 55) = 16.02, *p* < .001, ${\upeta }_{\text{p}}^{2} =$.23, and memory type, *F*(1, 55) = 141.42, *p* < .001, ${\upeta }_{\text{p}}^{2} =$.72, as well as their interaction, *F*(1, 55) = 122.25, *p* < .001, ${\upeta }_{\text{p}}^{2} =$.69, were significant. The simple main effects analyses further showed that during the item test, extradecisional processes took marginally longer in the standard format than in the blocked format,* F*(1, 55) = 3.69, *p* = .060, ${\upeta }_{\text{p}}^{2} =$.06. In contrast, during the source test, participants in the standard format had shorter nondecisional time compared to those in the blocked format, *F*(1, 55) = 69.87, *p* < .001, ${\upeta }_{\text{p}}^{2} =$.56. Overall, item responses were slowed down while source responses sped up in the standard format, and this seems not only due to response caution (threshold separation) but also due to nondecisional factors.

## Discussion

The current research examined the temporal aspect of item and source processing in the standard sequential source-monitoring test by comparison with a blocked testing procedure. We collected source decisions for recognized trials either in immediate succession to item decisions as in the standard format or in a separate test block upon the completion of the item tests. The goal was to elucidate whether item and source processing are executed in sequence consistent with the order of standard testing (i.e., first item processing, then source processing) or whether there can be (partial) temporal overlap between item and source processing during the item test of the standard format. To disentangle latent processes merged in raw RTs, while also considering accuracy, we applied the diffusion model (Ratcliff, 1978) analysis for each condition. Focusing primarily on the absolute drift rates, we compared the item and source decision speeds in the standard format with the blocked format to test the alternative time-courses. Although decision criteria and extradecisional processes showed differential effects across conditions, item and source decision speeds did not significantly differ by test format. On the behavioral level, participants knowing that they would be tested with an ensuing source test next upon their item decisions (i.e., an “old” response) gave slower item (but faster source) responses compared to those of the blocked format. However, these changes in RTs were unrelated to the speed of source information accumulation itself. Rather, our results point out response caution as a decision-level phenomenon and further underline the involvement of factors outside the decision process.

Given our preregistered hypotheses upon the interaction test of memory type and test format for the drift rates, we tentatively infer that there is no convincing evidence for temporal overlap between item and source. Accordingly, our results favored seriality, meaning that participants did not retrieve source information in parallel to item information when they were tested for their item memory. Otherwise, the transfer of information accumulation from the source test of the standard format to its item test should have led to a cost in information accumulation in the item test, and this should have been further supported by the reversed pattern in source decision speed.

The next question that could arise is why changes (or no considerable changes) especially in item decision speed across different test formats indicate whether source processing is taking place in parallel or not. This question can be theoretically discussed benefiting from the underlying mechanisms that subserve item and source memory (i.e., familiarity- vs. recollection-driven processes). It is particularly noteworthy that in episodic memory research, there is an ongoing debate on the contributions of these operating mechanisms to item and source memory, and, importantly, these different assumptions also invite different interpretations of the current results. We thus additionally address an alternative explanation. On the one hand, arguing from the dual-process accounts of recognition (Yonelinas, 1999), one could claim that source memory primarily relies on non-automatic resource-consuming recollection, whereas item memory is merely familiarity-based, that is, driven by automatic processes of memory. Borrowing from this assumption, the reasoning behind our preregistered hypotheses can be, admittedly, argued against. By its common definition, automaticity refers to the aspects that are fast, governed by stimuli (not deliberate intentions), and less effortful (cf. Spaniol & Bayen, 2002). If we had observed significant changes in item decision speed in the standard format, it might have supported the notion that the serially following source response is interfering with prior item retrieval. However, the inference is less clear when we do not observe a significant change as in the current situation. One would intuitively assume that any parallel processing, if it occurs, is not shown by item decision speed because this might be an efficient processing without costs to the time spent on the item test. On the other hand, from the source-monitoring framework (Johnson et al., 1993), it is legitimate to assume that recollection also contributes to item memory. Empirical dissociations of item and source memory (e.g., Bayen et al., 1996; Lindsay & Johnson, 1991) do not necessarily imply that there is no resource dependency at all. Johnson (2005) emphasizes that other episodic memory tasks, such as item recognition, involve some processes that are not unique to source memory. In fact, item memory is not familiarity-based only, but rather, additionally requires the recollection of the experimental context (e.g., “Is this specific test item from the learning episode?”). More specifically, although recollection demands of item memory are not as specific as source memory, item recognition may not be effortless. Therefore, bearing on the role of slower recollection processes (e.g., Hintzman & Caulton, 1997; McElree et al., 1999), one could rightfully claim that if there was other processing taking place in parallel, it should have changed the item decision speed. Likewise, the start of – primarily recollection-based – source decisions in the item test should have been further represented by higher source drift rates in the standard format compared to the blocked format, but this prediction did not hold, either.

Thus, the current findings cannot present decisive evidence to distinguish between serial versus parallel processing of item and source because they cannot discard alternative time-course scenarios independent from the assumptions about the nature of the mechanisms underlying item and source decisions. However, the current results interestingly enough do show that in the often-employed standard source-monitoring task querying about the source following “old” responses on the item test does not necessarily change the speed of item information accumulation on this previous test. In other words, item information accumulation is robust to an ensuing source query. This means that item information accumulation can be well studied within a source-monitoring paradigm. Both of theoretical (regarding its automaticity status) and practical interest, it will be interesting to further test this robustness of item information accumulation under different conditions (e.g., different encoding conditions (cf. Lindsay & Johnson, 1991) or different test designs (cf. Fox & Osth, 2022)). Further, it seems important to study whether people can engage in source accumulation parallel to item accumulation and whether this may bring benefits to source memory. In continuing this research, we deem it important to conceive the time course of item and source processing may not be limited to the dyadic description (i.e., seriality vs. parallelism), but rather, the overlap possibly can vary along a continuum, which may even manifest differently under certain conditions or for different individuals (e.g., the differential age-related deficits on item and source memory (cf. Old & Naveh-Benjamin, 2008) may also alter their time-courses).

Drift rates are able to capture the changes on the memory tasks of interest (e.g., see Spaniol et al., 2006, for dissociating semantic and episodic memory drift rates), and thus can be quite informative for the temporal ordering of item and source processing with appropriate designs. Notably, McKoon and Ratcliff (2012) defined the drift rate in the memory domain as “the quality of the evidence from memory that drives the decision process” (p. 417). However, as also acknowledged by Spaniol et al. (2006), the diffusion model alone is incapable of addressing which component(s) of memory (i.e., encoding, maintenance (vs. forgetting), retrieval) “the quality” maps on. Despite its utility in summarizing latent processes (e.g., the rate of evidence accumulation), it is agnostic with regard to the explanations of these processes (e.g., underlying mechanisms that generate evidence accumulation in a memory task; for further discussion, see Osth, Jansson et al., 2018b). This important constraint can be enhanced by integrated models of memory and decision-making (e.g., adopting characteristics of memory representations together with the assumptions of accumulator models; see Cox & Shiffrin, 2017; Nosofsky et al., 2011; Osth et al., 2023). In particular, such a combined model framework has the potential to decompose the drift rate into properties that are relevant to theories of memory.

Another important finding worthy of further attention is that we observed substantial effects on threshold separation, which gives further insight into the decision process underlying item and source responses. That is, the amount of information considered in decision making was significantly different between the test formats both for the item and source test. Compared to the blocked format, anticipating an immediate source test made the participants more conservative (i.e., setting higher thresholds) while making their item decisions. This difference was in the opposite direction for the source test, with smaller threshold separations in the standard format. This supports the notion that already being in the item recognition state requires less information to decide for a source response (cf. Tanyas & Kuhlmann, 2023). Technically speaking, prior item retrieval may just facilitate source attributions, thus leading to a closer distance between thresholds in the source test of the standard format, meaning that a smaller amount of evidence is necessary to accumulate in order to reach a threshold (i.e., make a source decision).

Overall, the distinct patterns in threshold separation suggests that our test format manipulation did really affect the decisional components underlying the item and source responses. Apart from threshold separation, further analyses of nondecision time showed that the test format manipulation not only yielded differences in decision settings but additionally affected nondecision-related factors. For example, comparisons with the blocked format indicated that in the standard format, nondecision time marginally increased during the item test but drastically decreased during the source test. A decrease in nondecision time is not surprising for the source test of the standard format since the same stimulus was tested again immediately after the “old” response. Thus, encoding of the stimulus in the source test should not have entailed as much time as in the blocked format, and this observed difference was most likely driven by perceptual encoding. At the same time, the intermixed presentation of the item and source test trials in the standard format means that participants also had to frequently switch between the keys assigned to the item and source responses. As a result, (marginally) increased nondecision time during the item test of the standard format can be mainly attributed to task preparation (cf. Schmitz & Voss, 2012) and motor activity – albeit being difficult to resolve precisely. It is thus clear that there are other factors underlying our test format manipulation that affect the speed of the responses without affecting the processing of the decision itself. The prominence of the drift rate enabled us to interpret the results that were corrected for nondecisional factors. Otherwise, it was likely to observe the effects hidden in the mean (or median) RTs or confounded with related accuracy.

As a limitation of our study, we must acknowledge that the trial number in our dataset, albeit being typical for source-monitoring tasks, is conventionally considered small for the diffusion model analysis (but also see other instances, e.g., Lerche, Neubauer, et al., 2018b). Yet, previous simulation research investigating parameter recovery reveals that under certain conditions the diffusion model can offer reliable results even with small trial numbers (Lerche et al., 2017). Moreover, it is not always desirable to increase the number of trials because this may increase involvement of other processes (e.g., guessing) as memory would be overtaxed, and responses would no longer primarily result from the accumulation process which the diffusion model is assumed to measure (Lerche, Christmann, et al., 2018a). Here, we showed a successful application of the diffusion model to the source-monitoring paradigm by adopting the typical circumstances. In addition, our application shows that the diffusion model is applicable to a higher-order cognition which subsumes multiple processes, as is the case for the current source-monitoring study (see also Lerche, Christmann, et al., 2018a). We recommend episodic memory researchers to consider the feasibility and benefit of the diffusion-modeling approach, especially in RT studies. Finally, we acknowledge that our modeling approach implemented here is limited to separate treatment of the item and source RTs. While more sophisticated modeling techniques could simultaneously treat both test RTs and also model the cognitive processes involved in the source attribution (e.g., see supplemental MPT analyses in the OSM), we think that our experimental approach of manipulating and comparing multiple test condition RTs is insightful with the benefit of being a quite direct approach to studying this research question. Comparing response speed across experimental conditions to learn about seriality versus parallelism of processing has a long-standing tradition in cognitive psychology (Sternberg, 1969). Although, as discussed, one might argue whether strict seriality can indeed be inferred from our results, our drift rate analysis clearly shows that querying for the source right after the item response (as is standard in source monitoring research) does not change the core item processing. More sophisticated model-based analyses linking response speed to memory parameters would require making much more arguable assumptions about the nature of memory processes involved in source monitoring, which are heavily debated (cf. Fox & Osth, 2022).

## Conclusion

Our study suggests that presenting item and source tests consecutively or in separate test blocks changed both decisional and nondecisional aspects of the item and source test responses. For the item response, we found a need for more information to decide and (marginally) increased nondecision time, resulting in a slower response in the standard format compared to the blocked format. However, when the source was queried immediately upon item detection, participants required less information for their source decisions and reduced nondecision time at this stage. Most importantly, although the way item and source memory are being tested affected other processes that confound overall RTs, the decision speeds matched with the drift rates did not change considerably across the test formats. A null effect in this interaction can be attributable to the lack of evidence for the temporal overlap of item and source processing, but it is also not warranted to assume a sharp separation between item and source retrieval even though they were probed in that order by the standard testing. Methodological progress can be made with more diagnostic models to rigorously assess direct connections between drift rates and memory characteristics (e.g., benefiting from process models). However, theoretical progress would likely come from the grounded assumptions of the source-monitoring framework, when the time-course question is reconciled more with the contributions of the different processes subserving item and source memory.

## Electronic supplementary material

Below is the link to the electronic supplementary material.

Supplementary file1 (DOCX 22 KB)

## Data Availability

The datasets analyzed during the current study are available in the Open Science Framework repository and can be accessed at: https://osf.io/j9zwr/.

## Footnotes

1 Interested readers can find the raw data as well as the dataset including individual estimates of the diffusion model parameters and the behavioral variables (i.e., accuracy rate and mean RTs) per participant in the OSF.

2 Note that we carefully considered performance-based accuracy as an initial step by calculating hits and false alarms on the item and source responses and excluding participants performing with poor accuracy (see Participants section). Thus, for the exclusions, accuracy in this study was assessed within the scope of source-monitoring perspective as summary measures on the participant-level. However, these were lenient criteria, and in our included dataset, there were still 14 participants whose item and/or source drift rates were negative, which indicates that their decision usually ended on the incorrect threshold. Put differently, their answers were not mostly driven by memory. We repeated our analyses without these participants for tapping into a closer inspection of memory-driven data. Of course, remaining participants’ answers were not guessing-free either, but an exclusion of those 14 participants can be seen as a proxy to memory. Findings yielded similar patterns. Regarding drift rates, neither the main effects of test format nor memory type, *F*s < 1, nor their interaction were significant, *F*(1, 41) = 1.62, *p* = .210, ${\upeta }_{\text{p}}^{2} =$.04.

## Code availability

The analysis codes and experiment scripts can be accessed via the Open Science Framework at: https://osf.io/j9zwr/.
