# Supplementary material for: Information accumulation on the item versus source test of source monitoring: Insights from diffusion modeling
Source: Mem Cognit. 2024 Oct 3;53(4):1124–39. doi: 10.3758/s13421-024-01636-2 (PMC12141409; doi:10.3758/s13421-024-01636-2)
Supplement: Supplementary file 1 — Supplementary file1 (DOCX 22 KB) [file 13421_2024_1636_MOESM1_ESM.docx]

# **Supplementary Material**

We measured item memory, source memory, and guessing biases separately with the two-high-threshold multinomial processing tree model of source monitoring (2HTSM; Bayen et al., 1996). Based on response frequencies across participants for each item type, the parameters of the 2HTSM represent latent cognitive processes with the probability estimates (see Erdfelder et al., 2009, for a general overview of MPT models). The specific submodel that we used here (see Bayen et al., 1996, for a detailed overview of alternative model versions) describes source-monitoring processes with four parameters: *D* (item memory with the assumption of equal detection of items presented on the top or bottom and new distractor items), *d* (source memory with the assumption of equal probability of remembering the top or bottom source), *b* (probability of guessing that an item is old), *g* (probability of guessing source A, assuming equal source guessing when source memory fails independent of item recognition status). Using the *multiTree* program (Moshagen, 2010), we fit a joint MPT model (*N* = 57), which estimates source-monitoring processes in each test format and allows for their comparisons across conditions. We assessed model fit via maximum likelihood estimation methods and the *G*^2^ statistic. For test of parameter differences across conditions, we relied on the χ^2^-distributed difference test statistic Δ*G*^2^. The most basic four-parameter submodel of the 2HTSM fit the data well, *G*^2^(4) = 5.53, *p* = .237. Table S1 shows parameter estimates by test format. Importantly, the fit of this restricted model assuming equal source memory for the top and bottom positions supports our aggregated analysis across spatial positions in the diffusion modeling presented in the main text.

To explore whether the accuracy results reported in the main text stem from memory and/or guessing effects, we next restricted all four parameters to be equal across the two conditions to test the effect of test format. Note that we did not indicate any specific hypotheses about the parameter differences across the test formats, so here we rather investigated them as exploratory. Restricting parameter *D* (i.e., item memory) significantly decreased the model fit, Δ*G*^2^(1) = 4.68, *p* = .030, indicating that item memory differed significantly by test format. We observed that item memory was higher in the blocked format than in the standard format (e.g., Tanyas & Kuhlmann, 2023). The immediate source test might have altered the characteristics of old-new recognition accuracy in the standard format (cf. Mulligan et al., 2010). However, when we tested the effect of test format on parameter *b* (i.e., item guessing), we found no significant difference, Δ*G*^2^(1) = 2.79, *p* = .095. Restrictions on parameter *d* (i.e., source memory), Δ*G*^2^(1) = 0.60, *p* = .437, and parameter *g* (i.e., source guessing), Δ*G*^2^(1) = 0.30, *p* = .582, did not significantly decrease the model fit, implying that neither source memory nor source guessing differed significantly by test format. Source guessing averaged across the test formats, *g* = .52, 95% CI [.50, .55], indicates a marginal bias to guess top, Δ*G*^2^(1) = 3.81, *p* = .051, but this tendency was comparable across the conditions. Crucially note that this is different from the starting point of the diffusion analysis on source RTs presented in the main text which did not code responses as top versus bottom but rather as correct versus incorrect.

## **Supplementary References**

Bayen, U. J., Murnane, K., & Erdfelder, E. (1996). Source discrimination, item detection, and multinomial models of source monitoring. *Journal of Experimental Psychology: Learning, Memory, and Cognition*, *22*(1), 197–215. <https://doi.org/10.1037/0278-7393.22.1.197>

Erdfelder, E., Auer, T.-S., Hilbig, B. E., Aßfalg, A., Moshagen, M., & Nadarevic, L. (2009). Multinomial processing tree models: A review of the literature. *Zeitschrift für Psychologie* / *Journal of Psychology*, *217*(3), 108–124. <https://doi.org/10.1027/0044-3409.217.3.108>

Moshagen, M. (2010). multiTree: A computer program for the analysis of multinomial processing tree models. *Behavior Research Methods*, *42*(1), 42–54. <https://doi.org/10.3758/BRM.42.1.42>

Mulligan, N. W., Besken, M., & Peterson, D. (2010). Remember-know and source memory instructions can qualitatively change old-new recognition accuracy: The modality-match effect in recognition memory. *Journal of Experimental Psychology, 36*(2), 558–566. <https://doi.org/10.1037/a0018408>

Tanyas, H., & Kuhlmann, B. G. (2023). The temporal development of memory processes in source monitoring: An investigation with mouse tracking. *Psychonomic Bulletin & Review*. <https://doi.org/10.3758/s13423-023-02289-z>

## **Table S1**

*Parameter Estimates and Confidence Intervals for Different Conditions of Test Format*

|  | Model parameters | | | |
| --- | --- | --- | --- | --- |
| Test format | *D* | *b* | *d* | *g* |
| Standard format | .38 [.35, .42] | .38 [.35, .41] | .54 [.45, .64] | .52 [.48, .55] |
| Blocked format | .44 [.40, .47] | .42 [.39, .45] | .59 [.51, .67] | .53 [.50, .56] |

*Note.* The presented model parameters are probability estimates that can range from 0 to 1. *D* = item memory; *b* = item guessing (chance level is .5); *d* = source memory; *g* = source guessing (estimates higher than the chance level of .5 indicate guessing bias towards “top”; estimates lower than .5 indicate guessing bias towards “bottom”). Brackets indicate 95% confidence intervals.
